# Radar HRRP Target Recognition Based on Stacked Autoencoder and Extreme Learning Machine

**DOI:** 10.3390/s18010173

**Published:** 2018-01-10

**Authors:** Feixiang Zhao, Yongxiang Liu, Kai Huo, Shuanghui Zhang, Zhongshuai Zhang

**Affiliations:** College of Electronic Science, National University of Defense Technology, Changsha 410073, China; zhaofeixiang14@nudt.edu.cn (F.Z.); huokai2001@163.com (K.H.); shzhang3@126.com (S.Z.); zhongshuai_fly@163.com (Z.Z.)

**Keywords:** radar target recognition, high-resolution range profile, deep learning, extreme learning machine, stacked autoencoder

## Abstract

A novel radar high-resolution range profile (HRRP) target recognition method based on a stacked autoencoder (SAE) and extreme learning machine (ELM) is presented in this paper. As a key component of deep structure, the SAE does not only learn features by making use of data, it also obtains feature expressions at different levels of data. However, with the deep structure, it is hard to achieve good generalization performance with a fast learning speed. ELM, as a new learning algorithm for single hidden layer feedforward neural networks (SLFNs), has attracted great interest from various fields for its fast learning speed and good generalization performance. However, ELM needs more hidden nodes than conventional tuning-based learning algorithms due to the random set of input weights and hidden biases. In addition, the existing ELM methods cannot utilize the class information of targets well. To solve this problem, a regularized ELM method based on the class information of the target is proposed. In this paper, SAE and the regularized ELM are combined to make full use of their advantages and make up for each of their shortcomings. The effectiveness of the proposed method is demonstrated by experiments with measured radar HRRP data. The experimental results show that the proposed method can achieve good performance in the two aspects of real-time and accuracy, especially when only a few training samples are available.

## 1. Introduction

Radar target recognition based on high-resolution range profile (HRRP) has become a research hotspot due to the acquisition and processing of HRRP data being relatively easy [1,2,3,4,5,6,7]. However, the non-cooperative recognition [8,9] with limited training samples is a challenging task. In the non-cooperative situation, such as at the battle with time, the amount of data under the test is usually huge but the training data is limited. This is because the radar system cannot be guaranteed to detect and track the non-cooperative targets for a long period of time, which will cause HRRP data to be lost or not observed. Therefore, it is very important to study the generalization performance of the recognition model and obtain good recognition performance under conditions of fewer training samples.

It is generally known that feature extraction is a key step in radar target recognition. The quality of the extracted features determines the performance of target recognition. Therefore, many scholars [3,6,10,11,12,13,14,15] have spent a lot of effort studying the methods of HRRP feature extraction. In [3], the principal component analysis (PCA) subspace model is utilized to minimize the reconstruction error. The multitask learning truncated stick-breaking hidden Markov model (MTL TSB-HMM) proposed in [6] is used to characterize the fast fourier transform (FFT) magnitude features of HRRP. Some other researchers [10,11] have used complicated statistic models to extract features from HRRP that have specific physical meaning, such as the target size, the center of gravity, the number of peaks, and so on. By using the RELAX and other super-resolution algorithms, the precise location and intensity information of radar HRRP scatterers can be extracted [12,13]. Manifold learning is used in target recognition of radar HRRP to reduce the feature dimensions [14]. Dictionary learning is adopted in [15] to extract the noise-robust and highly discriminative features of the HRRPs. These methods can work well on some occasions, but all of them are shallow architectures that cannot effectively characterize the radar HRRP. What is more, the features are mostly artificially designed and they need to rely on the experience of the researchers. If we do not have sufficient prior knowledge, the extracted features would be incomplete. Therefore, how to automatically extract the deep abstract features that are beneficial for target recognition has become an important issue.

The deep learning [16] theory put forward by Hinton can effectively solve the above problem. The essence of deep learning is to construct a neural network containing multiple hidden layers to map the data in order to obtain the deep essential characteristics [17]. A deep belief network is used in [18] to solve the non-cooperative target recognition with an imbalanced training dataset. As an important component of the deep learning structure, the stacked autoencoder (SAE) plays an important role in unsupervised learning and nonlinear feature extraction and it has also been applied in many fields [19,20,21,22]. The discriminant deep autoencoders (DDAEs) proposed in [23] are used to enhance the recognition performance where there are few training samples. Stacked corrective autoencoders (SCAEs) are proposed in [24], which employ the average profile of each HRRP frame as the correction term. In [25], a novel robust variational autoencoder model (RVAE) is proposed to explore the latent representations of HRRP. In these applications, SAE is used for feature learning to obtain the hierarchical abstract representation of the target. In addition, to implement recognition, we need to add a classifier to the top encoding layer of SAE and softmax regression is usually chosen. The last step of training is to fine-tune the parameters of all layers to achieve the desired recognition performance. This process will take a lot of time. Replacing the softmax regression with the extreme learning machine (ELM) as a classifier can improve the training speed.

ELM [26] is a new learning algorithm for single hidden layer feedforward neural networks (SLFNs). Its network topology is the same as that of back propagation (BP) [27] neural networks. It is also composed of an input layer, a hidden layer, and an output layer. Although the network structure is the same, the training method of ELM is quite different from that of the BP [28,29,30]. The BP network needs to use gradient descent algorithms to solve the network weights through multiple iterations, while the ELM solves the output weights by randomly generating the input weights and hidden biases. ELM has been widely studied by many scholars [31,32,33,34,35,36] because of its characteristics of fewer training parameters, fast learning speed, and good generalization ability. The regularized ELM is studied in [31], and the experimental results show that the addition of a regularization term can enhance the robustness and generalization performance of ELM. An online sequential ELM (OS-ELM) is proposed in [32], which can learn the training data one by one or chunk by chunk. Error minimized extreme learning machine (EM-ELM) is studied in [33]. In [34], the researchers extend the ELM algorithm from the real domain to the complex domain and propose a fully complex ELM (C-ELM). The enhanced incremental extreme learning machine (EI-ELM) is also studied in [35]. ELM based on the kernel method [36] is faster and more generalized than the support vector machine (SVM) [37]. Because of its own advantages, ELM has been widely used in many aspects, such as image processing [38], clustering [39], traffic signal recognition [40], fault detection [41], and so on. However, the existing ELM [31,32,34,35,36,37,38,41] does not make better use of the target category information when dealing with the recognition tasks. To solve this problem, a regularized ELM based on target class information is proposed in this paper. Besides, due to the random selection of input weights and hidden biases, the ELM tends to need more hidden nodes to achieve better generalization performance [29,39,42], which makes the network structure complex. In this paper, SAE is used to optimize the input weights and hidden biases of ELM, which then achieves better results with fewer hidden layer nodes. The features of the proposed method are summarized as follows:

(a) The proposed model is “end to end”, the input is the original radar HRRP data, and the output is the target class.

(b) This paper proposes a combination of SAE and regularized ELM, which can improve the recognition performance by making full use of the advantages of SAE and ELM. Compared with the shallow learning algorithms such as PCA [3], MTL TSB-HMMs [6], ELM [26], and so on, the proposed algorithm can extract the inherent characteristics of the target. Since the network is not required to be fine-tuned, the proposed algorithm is faster than the other deep learning models [18,23,24,25].

(c) The proposed method does not only improve the training speed but also gets good performance when the training sample is small.

The rest of this paper is organized as follows: Section 2 introduces the relevant theoretical knowledge of SAE and ELM. In Section 3, we present the regularized ELM, then we also introduce the learning process of SAE-ELM. Experimental results are analyzed in Section 4, and in Section 5 the paper is summarized.

## 2. Theoretical Background

### 2.1. Description of HRRP

**Figure 1:**
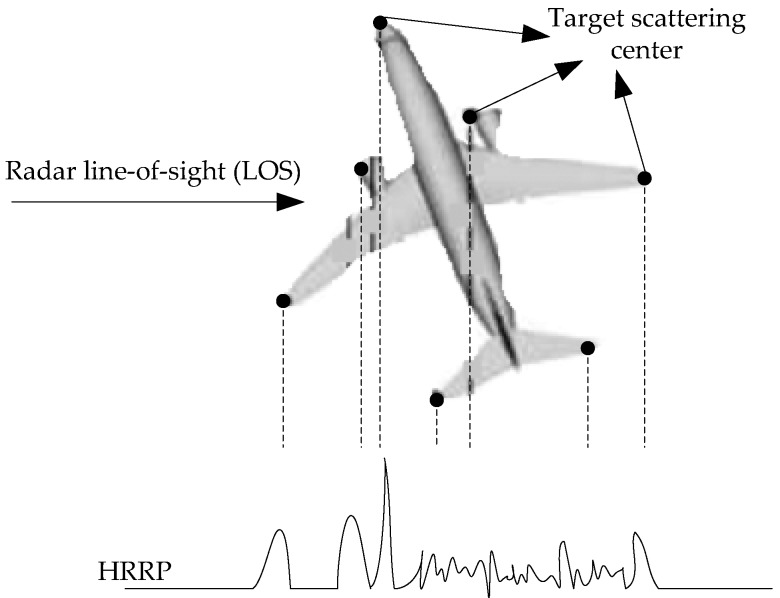
Illustration of a high-resolution range profile (HRRP) sample from a plane target.

HRRP can be regarded as the amplitude of the coherent summations of the complex time returns from target scatters in each range cell [3], which represents the projection of the complex returned echoes from the target scattering centers onto the radar line-of-sight (LOS) [4]. The illustration of an HRRP sample from a plane target is shown in Figure 1. Since HRRP contains the target-important structural features such as target size and the distribution of scattering centers, etc., radar HRRP target recognition has drawn much attention from the radar automatic target recognition community [3,4,5,6,7].

### 2.2. Stacked Autoencoder

**Figure 2:**
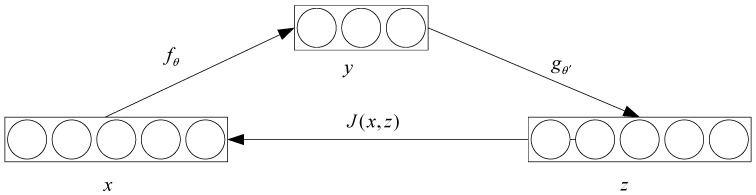
The structure of an autoencoder (AE).

An autoencoder (AE) is an unsupervised learning algorithm. Figure 2 shows a simple model structure for an AE:

Given an unlabeled dataset ${\left\{x^{\left(i\right)}\right\}}_{i=1}^{m}$, each of its training data $x^{\left(i\right)}$ is encoded by an encoder and the feature representation $y^{\left(i\right)}$ of the hidden layer can be obtained: $y^{\left(i\right)}=f_{\theta }(x^{\left(i\right)})=s(Wx^{\left(i\right)}+b)$ where θ=(W,b) is the network parameter, W is the weight matrix, b is the bias vector, and s(x) is the activation function; the sigmoid function is selected here and $s(x)=1/\left(1+e^{-x}\right)$. Then, the feature representation $y^{\left(i\right)}$ of the hidden layer is decoded by the decoder and the reconstruction vector $z^{\left(i\right)}$ can be obtained: $z^{\left(i\right)}=g_{\theta^{\prime }}(y^{\left(i\right)})=s(W^{\prime }y^{\left(i\right)}+b^{\prime })$, where $\theta^{\prime }=(W^{\prime },b^{\prime })$, $W^{\prime }$ is the weight matrix and $W^{\prime }=W^{T}$. In fact, the optimization of the model parameters is to minimize the reconstruction error [16]:

(1) $$\begin{array}{ll} \theta^{*},{\theta^{\prime }}^{*} & =\arg\min_{\theta ,\theta^{\prime }}\sum_{i=1}^{m}J(x^{\left(i\right)},z^{\left(i\right)})\\ & =\arg\min_{\theta ,\theta^{\prime }}\sum_{i=1}^{m}J(x^{\left(i\right)},g_{\theta^{\prime }}(f_{\theta }(x^{\left(i\right)}))) \end{array}$$

where m is the sample number and J is the cost function. The expression for J is $J(x,z)=\frac{1}{2}{\left\|z-x\right\|}^{2}$.

For a dataset containing m samples, the total cost function is:

(2) $$J_{1}=[\frac{1}{m}\sum_{i=1}^{m}\left(\frac{1}{2}{\left\|z_{i}-x_{i}\right\|}^{2}\right)]+\frac{\lambda }{2}\sum_{l=1}^{n_{l}-1}\sum_{i=1}^{s_{l}}\sum_{j=1}^{s_{l+1}}{\left(W_{ji}^{\left(l\right)}\right)}^{2}$$

where $W_{ji}^{\left(l\right)}$ is the connection weights between the *i*-th neurons of layer l and the *j*-th neurons of layer l+1; $n_{l}$ and $s_{l}$ indicate the number of network layers and the number of neurons of layer l, respectively. The first part of Equation (2) is a mean squared error term and the second part is a weight decay term, which can be seen as a way to compromise between the small weights and minimized cost function [21]. The second term of Equation (2) is intended to prevent overfitting [19].

If the number of hidden layer nodes is large, and even more than the number of input layer nodes, the sparsity constraint needs to be added on the hidden units [19]. Hidden units are constrained to be zero most of the time when the activation function is selected as a sigmoid function [43]. This is motivated by the structure of the brain in which most of the neurons are inactive most of the time. By forcing the hidden units to have mostly zero activations/values, interesting representations can be learned. Then, the overall cost function is expressed as follows:

(3) $$J_{s}=J_{1}+\eta \sum_{j=1}^{s_{2}}\mathrm{KL}(\rho \|{\hat{\rho }}_{j})$$

where the second part of Equation (3) represents the sparse penalty term and the penalty term used in this paper is based on Kullback-Leibler (KL) divergence [44]. KL indicates the relative entropy [24] between the two Bernoulli random variables with the mean of ρ and the mean of ${\hat{\rho }}_{j}$, and $\mathrm{KL}(\rho \|{\hat{\rho }}_{j})=\rho \log\frac{\rho }{{\hat{\rho }}_{j}}+(1-\rho )\log\frac{1-\rho }{1-{\hat{\rho }}_{j}}$. If ${\hat{\rho }}_{j}=\rho$, $\mathrm{KL}(\rho \|{\hat{\rho }}_{j})$ reaches the minimum value of 0, and if ${\hat{\rho }}_{j}$ approaches 0 or 1, the $\mathrm{KL}(\rho \|{\hat{\rho }}_{j})$ increases dramatically. $s_{2}$ is the number of neurons in the hidden layer. η is the weight of the sparsity penalty.

**Figure 3:**
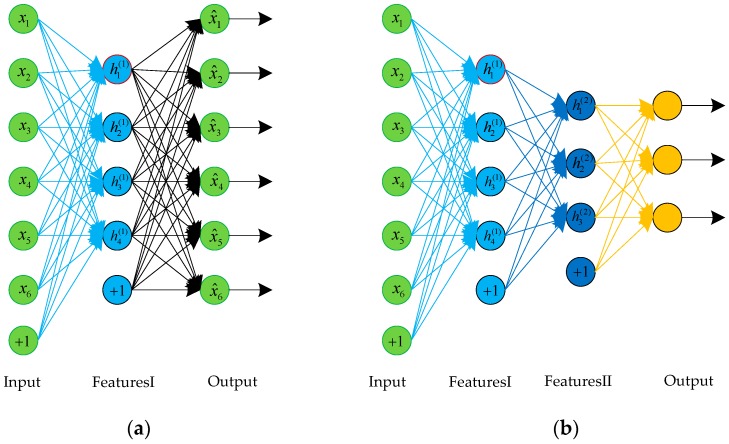
The structure of an AE and a stacked autoencoder (SAE). (**a**) A three-layer AE; (**b**) An SAE composed of two autoencoders.

SAE is a neural network consisting of multiple layers of autoencoders, and the structure of an SAE is shown in Figure 3. We can use a greedy layer-wise training method to train SAE; that is, the output of each layer is wired to the input of the successive layer. Then, the BP algorithm is used to fine-tune the whole network.

### 2.3. Extreme Learning Machine

Given a set of N training datasets $\left(x_{i},t_{i}\right)$ where i=1,2,⋯,N, $x_{i}={\left[x_{i1},x_{i2},\cdots ,x_{in}\right]}^{T}\in R^{n}$, and $t_{i}={\left[t_{i1},t_{i2},\cdots ,t_{im}\right]}^{T}\in R^{m}$, $x_{i}$ is an n-dimensional input vector and $t_{i}$ is the expected output. The output function of ELM with L hidden nodes is represented as follows:

(4) $$\sum_{i=1}^{L}\beta_{i}g(w_{i}\cdot x_{j}+b_{i})=o_{j}\;j=1,2,\cdots ,N$$

where $w_{i}={\left[w_{i1},w_{i2},\cdots ,w_{in}\right]}^{T}\in R^{n}$ is the weight vector of input nodes to hidden nodes and $b_{i}$ is the bias of *i-th* hidden node; $\beta_{i}=[\beta_{i1},\beta_{i2},\cdots ,\beta_{im}]\in R^{m}$ is the weight vector between hidden nodes and the output nodes; g(x) is the activation function of the hidden layer; and $o_{j}$ is the output vector.

If the SLFNs with L hidden nodes can approximate the N samples with zero error, we know that Equation (4) can be converted to the following formula [26,45]:

(5) $$\left\{ \begin{array}{l} \sum_{i=1}^{L}\beta_{i}g(w_{i}\cdot x_{1}+b_{i})=t_{1}\\ \;\vdots \\ \sum_{i=1}^{L}\beta_{i}g(w_{i}\cdot x_{N}+b_{i})=t_{N} \end{array}\right.$$

The above equations can be written as:

(6) Hβ=T

where

(7) $$\begin{array}{l} H(w_{1},\cdots ,w_{L},b_{1},\cdots ,b_{L},x_{1},\cdots ,x_{N})=\\ {\left[\begin{array}{ccc} g(w_{1}\cdot x_{1}+b_{1}) & \cdots & g(w_{L}\cdot x_{1}+b_{L})\\ \;\vdots & \cdots & \;\vdots \\ g(w_{1}\cdot x_{N}+b_{1}) & \cdots & g(w_{L}\cdot x_{N}+b_{L}) \end{array}\right]}_{N\times L} \end{array}$$

(8) $$\beta ={\left[\begin{array}{l} \beta_{1}^{T}\\ \;\vdots \\ \beta_{L}^{T} \end{array}\right]}_{L\times m}\;T={\left[\begin{array}{l} t_{1}^{T}\\ \;\vdots \\ t_{N}^{T} \end{array}\right]}_{N\times m}$$

So training the SLFNs corresponds to finding the norm least-squares solution $\hat{\beta }$, which can be shown as follows:

(9) $$\hat{\beta }=H^{+}T$$

where $H^{+}$ is the Moore–Penrose generalized inverse [46,47] of hidden layer output matrix H.

Then, Equation (9) can be converted to:

(10) $$\beta =\left\{ \begin{array}{ll} {\left(\frac{I}{C}+H^{T}H\right)}^{-1}H^{T}T, & N>L\\ H^{T}{\left(\frac{I}{C}+HH^{T}\right)}^{-1}T, & N<L \end{array}\right.$$

where I is the unit matrix and C is the regularization coefficient.

ELM can also be explained using the optimization method. The ELM theory aims to reach the smallest training error ${\left\|H\beta -T\right\|}^{2}$ and the smallest norm of the output weights ‖β‖ [28,31]. Then, the solution of Equation (6) can be obtained by:

(11) $$\begin{array}{l} \mathrm{Minimize}:\;L_{P_{ELM}}=\frac{1}{2}{\left\|\beta \right\|}^{2}+C\frac{1}{2}\sum_{i=1}^{N}{\left\|\xi_{i}\right\|}^{2}\\ \mathrm{Subject}\;\mathrm{to}:\;h(x_{i})\beta =t_{i}^{T}-\xi_{i}^{T},\;i=1,\cdots ,N \end{array}$$

where $\xi_{i}$ is the training error vector of the m output nodes corresponding to training sample $x_{i}$, and $h(x_{i})$ is the hidden layer output vector of *i*-th sample $x_{i}$. According to the Karush–Kuhn–Tucker (KKT) theorem [48], the same solution as Equation (10) can be obtained.

Thus, the learning steps of the ELM can be summarized as Algorithm 1:
**Algorithm 1: ELM**Input: training sets $\left\{x_{i},t_{i}\right\}$, ($x_{i}\in R^{n}$, $t_{i}\in R^{m}$, i=1,2,⋯,N), activation function g(x) and hidden nodes L.Output: output weight vector β(1): Set random values to the input weights $w_{i}$ and the hidden layer biases $b_{i}$;(2): Calculate the hidden layer output matrix H according to Equation (7);(3): Calculate the output weight vector β according to Equation (9).

## 3. Stacked Autoencoder-Regularized Extreme Learning Machine

As we know the sample data has similar attributes and distribution features, we can use the similar relationships to enhance the generalization performance of ELM. Therefore, in this section, we propose a regularized ELM based on the class information of the target. Optimizing the output weights by maximizing the within-class scatter degree and by minimizing the inter-class scatter degree can make the ELM have better recognition and generalization ability. In addition, due to the random selection of input weights and hidden biases, ELM tends to need more hidden nodes to achieve better generalization performance, which makes the network structure complex. To address this issue, SAE is used to optimize the input weights and hidden biases of ELM; this achieves better results with fewer hidden layer nodes.

### 3.1. Regularized ELM Based on the Class Information of the Target

Given a set of sample sets $\left\{x_{i}^{\left(j\right)};j=1,2,\cdots ,c;i=1,2,\cdots ,n_{j}\right\}$, the number of classes is c and the $\omega_{j}$ class contains $n_{j}$ samples. The inter-class scatter matrix of class $\omega_{j}$ is defined as

(12) $$S_{W}^{\left(j\right)}=\frac{1}{n_{j}}\sum_{i=1}^{n_{j}}(x_{i}^{\left(j\right)}-m_{j}){\left(x_{i}^{\left(j\right)}-m_{j}\right)}^{T},\;j=1,2,\cdots ,c$$

where $m_{j}$ is the mean of $\omega_{j}$ class samples and $m_{j}=\frac{1}{n_{j}}\sum_{i=1}^{n_{j}}x_{i}^{\left(j\right)},\;j=1,2,\cdots ,c$.

The total inter-class scatter matrix is defined as

(13) $$S_{W}=\sum_{j=1}^{c}\frac{n_{j}}{N}S_{W}^{\left(j\right)}$$

The within-class scatter matrix is defined as

(14) $$S_{B}=\sum_{j=1}^{c}\frac{n_{j}}{N}(m_{j}-m){\left(m_{j}-m\right)}^{T}$$

where m is the mean of all samples and $m=\frac{1}{N}\sum_{i=1}^{N}x_{i}$.

To improve the recognition performance, we should maximize the within-class scatter matrix and minimize the inter-class scatter matrix [49]. Therefore, we define the matrix S as shown below:

(15) $$S=\frac{S_{W}}{\tau S_{B}}\;(0<\tau \leq 1)$$

Then, the optimization formula of regularized ELM can be written as:

(16) $$\begin{array}{l} \mathrm{Minimize}:\;L_{P_{ELM}}=\frac{1}{2}\beta^{T}S\beta +C\frac{1}{2}\sum_{i=1}^{N}{\left\|\xi_{i}\right\|}^{2}\\ \mathrm{Subject}\;\mathrm{to}:\;h(x_{i})\beta =t_{i}^{T}-\xi_{i}^{T},\;i=1,\cdots ,N \end{array}$$

We can solve the above problem by defining the Lagrange function:

(17) $$\min:\;L_{ELM}=\frac{1}{2}\beta^{T}S\beta +C\frac{1}{2}\sum_{i=1}^{N}{\left\|\xi_{i}\right\|}^{2}-\sum_{i=1}^{N}\sum_{j=1}^{m}\alpha_{i,j}(h(x_{i})\beta_{j}-t_{i,j}+\xi_{i,j})$$

then

(18) $$\begin{array}{l} \frac{\partial L_{ELM}}{\partial \beta_{j}}=0\to \beta S=H^{T}\alpha \\ \frac{\partial L_{ELM}}{\partial \xi_{i}}=0\to \alpha_{i}=C\xi_{i},\;i=1,\cdots ,N\\ \frac{\partial L_{ELM}}{\partial \alpha_{i}}=0\to h(x_{i})\beta -t_{i}^{T}+\xi_{i}^{T}=0,\;i=1,\cdots ,N \end{array}$$

where $\alpha_{i}={\left[\alpha_{i,1},\cdots ,\alpha_{i,m}\right]}^{T}$ and $\alpha ={\left[\alpha_{1},\cdots ,\alpha_{N}\right]}^{T}$.

Then, the solution to Equation (16) is:

(19) $$\beta =\left\{ \begin{array}{ll} {\left(\frac{S}{C}+H^{T}H\right)}^{-1}H^{T}T, & N>L\\ H^{T}{\left(\frac{S}{C}+HH^{T}\right)}^{-1}T, & N<L \end{array}\right.$$

Thus, the learning steps of the regularized ELM can be summarized as Algorithm 2:
**Algorithm 2: Regularized ELM**Input: training sets $\left\{x_{i},t_{i}\right\}$, ($x_{i}\in R^{n}$, $t_{i}\in R^{m}$, i=1,2,⋯,N), activation function g(x) and hidden nodes L.Output: output weight vector β(1): Calculate $S_{W}$ and $S_{B}$, then calculate S according to Equation (15);(2): Set random values to the input weights $w_{i}$ and the hidden layer biases $b_{i}$;(3): Calculate the hidden layer output matrix H according to Equation (7);(4): Calculate the output weight vector β according to Equation (19).

### 3.2. SAE-ELM

**Figure 4:**
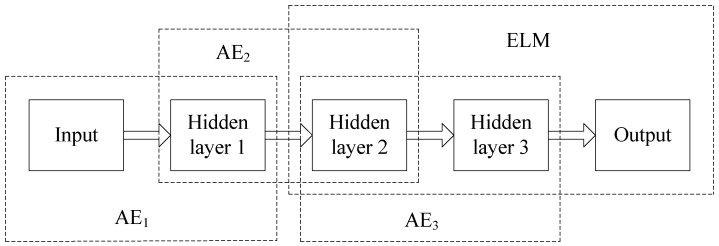
The stacked autoencoder-extreme learning machine (SAE-ELM) system architecture.

**Figure 5:**
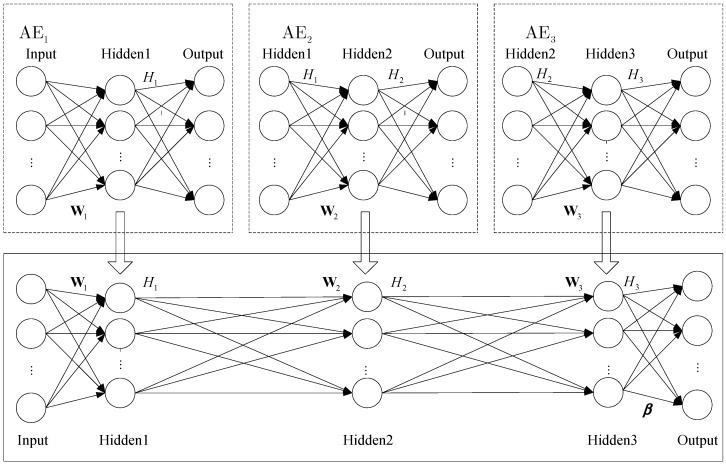
Illustration of the structure of SAE-ELM.

In order to implement recognition, we need to add a classifier to the top encoding layer of SAE. In this section, we propose that using ELM instead of softmax as a classifier can effectively improve the network training speed. In addition, we can get the appropriate ELM network parameters by training SAE. The SAE–ELM system architecture is shown in Figure 4, and the illustration of the structure is shown in Figure 5. The learning process of SAE–ELM is as follows:

(1) Establish the first layer of AE network and, as described in Section 2.2, use the gradient descent method to train the network. Then, we can obtain the output $H_{1}$ of the first hidden layer and the network parameters $\theta_{1}$. $H_{1}$ is the characteristic representation of the input data and $\theta_{1}=(W_{1},b_{1})$.

(2) Establish the second layer of the AE network. The first layer output $H_{1}$ is input as the second layer. We use the gradient descent method to train the network. Then, the output $H_{2}$ of the second hidden layer and the network parameters $\theta_{2}$ are available and $\theta_{2}=(W_{2},b_{2})$.

(3) Establish the third layer of the AE network to determine the parameters of ELM. ELM not only has a faster learning speed than the traditional learning methods but it also has a good generalization performance. However, ELM needs more hidden nodes than conventional tuning-based learning algorithms due to the random set of input weights and hidden biases. Therefore, we establish the third layer of the AE network to determine the input weights and hidden biases for ELM. Similar to step (2), the output $H_{3}$ of the third hidden layer and network parameters $\theta_{3}=(W_{3},b_{3})$ can be obtained. We can utilize $W_{3}$ as the input weights and $b_{3}$ as the hidden biases of ELM, then the hidden layer output matrix of ELM is $H_{3}$.

(4) Establish the ELM network as a classifier. The input is $H_{2}$, the input weights and hidden biases are $\theta_{3}=(W_{3},b_{3})$, and the hidden layer output matrix is $H_{3}$. Then, as described in Section 3.1, the output weight vector β can be calculated according to Equation (19).

## 4. Experimental Results and Discussion

In this section, we will verify the effectiveness of the proposed algorithm. The experiments were performed on an Intel(R) Core(TM) 3.60 GHz CPU with 8 GB of RAM and the MATLAB R2013a environment.

**Figure 6:**
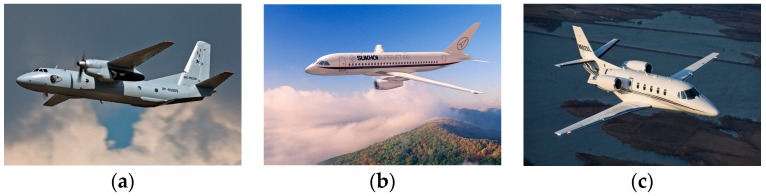
The Imagery targets. (**a**) An-26 airfreighter; (**b**) Yark-42; (**c**) Citation business jet.

In this section, we utilize measured radar HRRP data from three real airplanes that are measured by a C-band radar with a center frequency of 5.52 GHz and a bandwidth of 400 MHz to validate the effectiveness of the proposed method. The An-26 is a medium-sized propeller airplane, the Yark-42 is a large and medium-sized jet airplane, and the Citation business jet is a small-sized jet airplane. The three aircraft models are shown in Figure 6. The detailed size of each airplane and the parameters of the measured radar are listed in Table 1. In our experiments, each aircraft target has 26,000 HRRP samples and the measured HRRP is a 256-dimensional vector.

**Figure 7:**
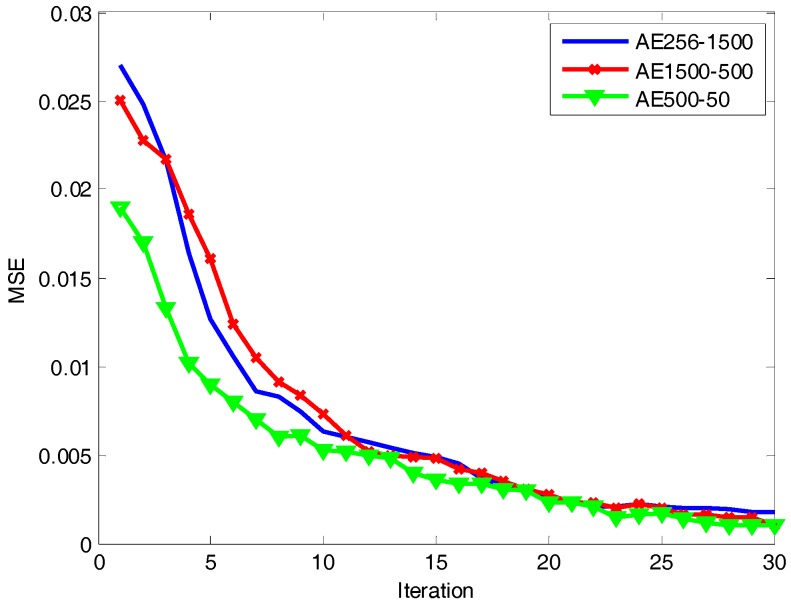
Reconstruction error of SAE pre-training.

In order to verify the validity of the algorithm proposed in this paper, we compared it with the commonly used methods: PCA [3], MTL TSB-HMMS [6], ELM [26], SAE [21], and DDAEs [23]. The activation function of the hidden layer of ELM is sigmoid and G(a,b,x)=1/(1+exp(−(a⋅x+b))). The regularization coefficient C is 0.2. The number of hidden nodes of ELM is 1500. Due to the sample dimension being 256, we set the number of nodes in the visible layer of deep architecture to 256. It is well known that a more abstract feature representation can be obtained with an increase in the network depth. However, too many layers can make the network difficult to train effectively and brings in more parameters to learn. Through the analysis of the experimental data and task requirements, we found that three is a good choice for the number of hidden layers. Therefore, we set the number of hidden layers to be three and the number of nodes in the hidden layers as 1500-500-50, respectively. From Figure 7 we can see that the mean square error (MSE) of each layer reconstruction of the network model decreases with an increase of iterations. When the number of iterations is 25, the MSE is less than 0.003. Therefore, in order to speed up the training, we set the number of iterations in the network to 25.

Before network training, data pre-processing is needed to solve the amplitude-scale and time-shift sensitivities. According to the previous study [3,5,6,7], we usually use the energy normalization method and time-shift compensation algorithm to cope with the above issues.

**Figure 8:**
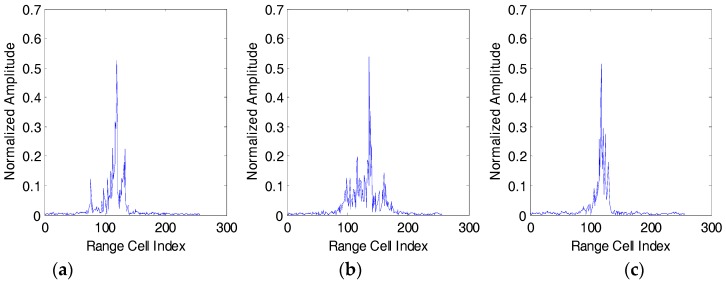
Range profiles of the three real airplanes. (**a**) An-26 airfreighter; (**b**) Yark-42; (**c**) Citation business jet.

Figure 8 shows the range profiles of pre-processed aircraft targets. In the non-cooperative situation, such as at the battle with time, the amount of data under the test is usually huge, but the training data is limited. This is because the radar system cannot be guaranteed to detect and track the non-cooperative targets for a long period of time, which will cause HRRP data to be lost or not observed. Therefore, it is very important to study the generalization performance of the model and obtain good recognition performance under the conditions of fewer training samples.

**Figure 9:**
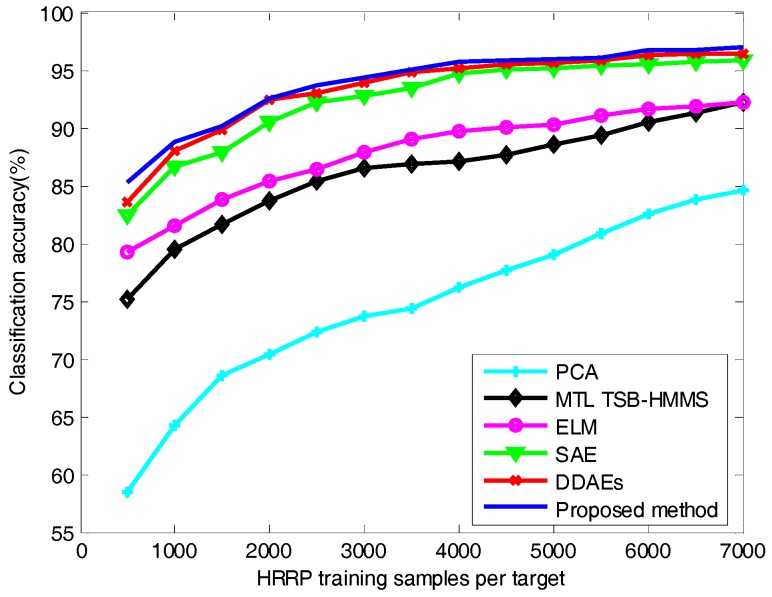
Classification accuracy against different HRRP training samples.

As is shown in Figure 9, as the number of training samples increases, the classification accuracy of different algorithms also increases. However, deep architecture algorithms (e.g., SAE, DDAEs, and the proposed method) are more accurate than shallow architecture algorithms (e.g., PCA, MTL TSB-HMMS, and ELM). The traditional recognition algorithms rely on the experience of the researchers and require a complete set of training samples to ensure excellent recognition performance. Because of the shallow architecture, these algorithms cannot effectively separate the intrinsic class information of the target from some external factors in the feature space. The depth structure algorithms lose the inherent class information of the target as little as possible while demodulating the coupling relationship between various factors layer by layer. More intuitively, the low-level features in the deep network are usually distributed and can be shared among different classes, while the high-level features are usually more abstract and more separable. Therefore, better generalization performance is a great advantage of deep networks. Due to the proposed method not only obtaining the deep feature representation of radar HRRP but also making better use of the target category information, the classification performance of the proposed method is better than that of SAE and DDAEs. In addition, when the training sample is smaller, the classification performance of the proposed method is better than the other algorithms, which shows that the proposed method has better generalization performance. When the number of training samples for each target is 3500, the classification accuracy of different algorithms is listed in Table 2. It can be seen from the table that when the number of training samples is 3500, the accuracy of the proposed algorithm reaches 95.01%, which is 0.22% higher than that of the DDAE algorithm, and 1.5% higher than that of the SAE. The accuracy of the shallow structure algorithms is not more than 90%. It can be concluded that the proposed method can obtain better classification performance when there is only a small amount of training samples available.

As shown in Table 3, the training time of SAE, DDAEs, and the proposed method are compared. The proposed method is almost five times faster than SAE in training time; that is because we need to add the softmax regression classifier to the top encoding layer of SAE, and the last step of training is to fine-tune the parameters of all layers to achieve the desired classification performance. This process will take a lot of time. The proposed method adds ELM with faster learning speed and less required tuning parameters to the top layer of SAE as a classifier. The proposed method does not need to fine-tune the parameters of all layers, thus reducing the network training steps and training time. SAE and DDAEs are similar in training time because their network structures are the same.

**Figure 10:**
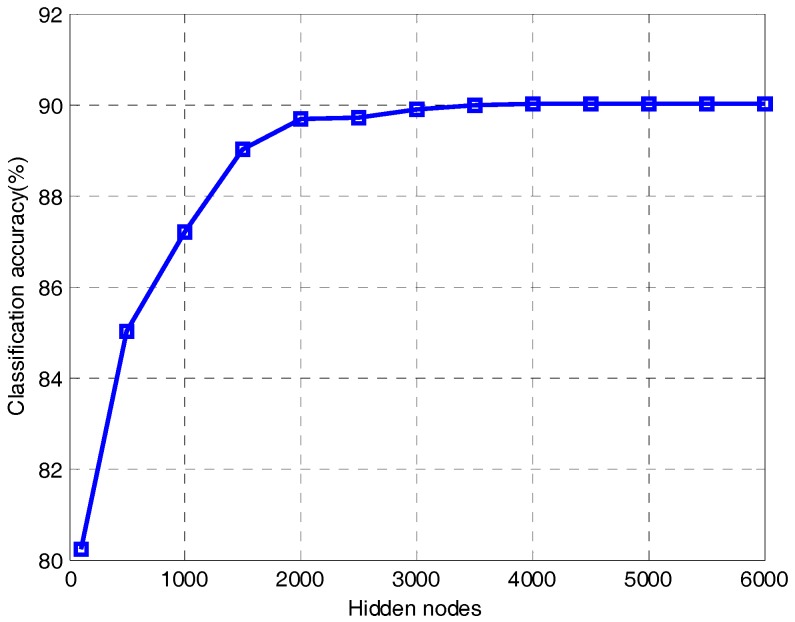
Accuracy of ELM in different hidden nodes.

It can be seen from Figure 10 that the classification accuracy of ELM becomes much better as the hidden nodes increase. When the number of hidden nodes is 1500, the classification accuracy is 89.01%. When the number of hidden nodes increases to 4000, ELM reaches an accuracy of 90.01%. After that, the value is almost unchanged all the time because the ELM is in an over-fitting state. Therefore, we know that in order to get a better classification effect, ELM needs more hidden nodes, which will make the network structure more complex. As we know from Table 2, only 50 hidden nodes are required to obtain an accuracy of 95.01% when the proposed method uses regularized ELM for classification. Therefore, the proposed method can effectively reduce the hidden nodes of ELM and simplify the network structure.

## 5. Conclusions

In this paper, we have proposed a novel radar HRRP target recognition method based on SAE and regularized ELM. SAE, as an important component of the deep learning structure, can extract deep features and mine the essential information of radar HRRP, which has a beneficial effect on recognition. ELM is also useful for recognition because of its fast learning speed and good generalization performance. Experimental results show that the proposed method does not only reduce the network training time but also makes the ELM achieve high recognition accuracy under the condition of using fewer hidden nodes. In addition, when there is only a small amount of training samples available, the proposed method can also obtain good recognition performance. However, we also know that in real situations the training samples are usually obtained under the condition of high signal-to-noise ratio (SNR) via some cooperative measurement experiments, while the test samples are usually achieved in the non-cooperative circumstance where the high SNR cannot be guaranteed due to the severe measurement conditions. Thus, it is important to optimize the proposed method to match the noise level of the received test samples in the recognition stage. Stacked denoising sparse autoencoder (sDSAE) can effectively eliminate the influence of noise. Therefore, in the near future, we will consider combining sDSAE with ELM to solve this problem.

## Figures and Tables

**Table 1:** Parameters of the airplanes and radar in the inverse synthetic aperture radar (ISAR) experiment.

Radar Parameters	Center Frequency		5520 MHz
	Bandwidth		400 MHz
Airplane	Length (m)	Width (m)	Height (m)
An-26	23.80	29.20	9.83
Yark-42	36.38	34.88	9.83
Citation business jet	14.40	15.90	4.57

**Table 2:** The classification accuracy comparison of different methods.

Method	Classification Accuracy (%)
PCA	74.38
MTL TSB-HMMS	86.87
ELM	89.01
SAE	93.51
DDAEs	94.79
Proposed method	95.01

**Table 3:** The training time of the different methods.

Method	Training Time (s)
SAE	624.73
DDAEs	625.14
Proposed method	106.67
